# Patientenveranstaltungen in der deutschen Urologie: Trend zu Hybridformaten?

**DOI:** 10.1007/s00120-023-02162-w

**Published:** 2023-08-11

**Authors:** Philipp Karschuck, Laura Müller, Christer Groeben, Cem Aksoy, Luka Flegar, Aristeidis Zacharis, Martin Baunacke, Christian Wülfing, Johannes Huber

**Affiliations:** 1 Klinik für Urologie, Philipps-Universität Marburg, Baldingerstraße, 35033 Marburg, Deutschland; 2 Klinik und Poliklinik für Urologie, Technische Universität Dresden, Dresden, Deutschland; 3 Klinik für Urologie, Asklepios Klinik Altona, Hamburg, Deutschland

**Keywords:** Hybridveranstaltung, Fachgesellschaften, Patienteninformation, Patientenforum, Versorgungsforschung, Hybrid event, Professional medical society, Patient information, Patient forum, Health services research

## Abstract

**Hintergrund und Fragestellung:**

Patientenveranstaltungen sind ein wichtiges Instrument, um auf das steigende Bedürfnis der Öffentlichkeit nach Gesundheitsinformationen zu reagieren. Hierfür bietet die Deutsche Gesellschaft für Urologie e. V. (DGU) auf ihren Jahreskongressen das „Patientenforum“ an. Ziel der Arbeit war die Evaluation der Veranstaltungen in den Jahren 2017 bis 2019 und ein Vergleich mit dem ersten digitalen Patientenforum 2020.

**Material und Methoden:**

Anhand eines zweiseitigen, standardisierten Fragebogens befragten wir die Besucher*innen der Präsenzpatientenforen (Präsenzgruppe = P) der drei Jahreskongresse der DGU 2017–2019 sowie die Nutzer*innen des digitalen Angebots 2020 (Onlinegruppe = O).

**Ergebnisse:**

Für die Jahre 2017–2019 erhielten wir *n* = 71 und für 2020 *n* = 18 Datensätze. Das mediane Alter der Besucher*innen lag bei 64 (Spannweite 30–89) Jahren. Männlich waren 66 % (P) vs. 83 % (O) der Teilnehmer*innen (*p* = 0,005). Das Angebot wurde von beiden Gruppen gleichermaßen insgesamt als gut bis sehr gut bewertet, d. h. in Schulnoten 1,6 (P) vs. 1,6 (O; *p* = 0,7). Die Möglichkeit Fragen zu stellen wurde entsprechend der geringeren Interaktion im digitalen Format in Schulnoten mit 1,5 (P) vs. 2,8 (O) schlechter bewertet (*p* = 0,003). Auf die Frage nach dem zukünftig gewünschten Veranstaltungsformat sprachen sich die Nutzer des digitalen Patientenforums mit zwei Dritteln für eine Hybridveranstaltung vor Ort und online aus.

**Schlussfolgerung:**

Patientenveranstaltungen eignen sich als Kommunikationsform für die Öffentlichkeit und werden von den Besucher*innen gut bewertet. Insbesondere die direkte Interaktion mit Expert*innen hat hier einen hohen Stellenwert. Präsenzformate sind mit einem hohen logistischen Aufwand sowie hohen Kosten verbunden und ihre Reichweite ist limitiert. Zukünftig können Hybridformate eine sinnvolle Alternative sein, da sie die Vorteile von Online- und Präsenzformaten kombinieren.

## Abstract (en)

**Background:**

Patient events are an important tool to respond to the increasing need of the public for health information. Through the “patient forum”, the German Society of Urology (DGU) is committed to patient information and public relations at its annual congresses. The goal of the study was to evaluate the events from 2017–2019 and to compare them with the first digital patient forum in 2020.

**Materials and methods:**

Using a two-page standardized questionnaire, we surveyed the visitors of the presence patient forums (presence group = P) of the three annual congresses of the DGU 2017–2019 as well as the users of the digital event 2020 (online group = O).

**Results:**

We obtained 71 records for 2017–2019 and 18 for 2020. The median age of visitors was 64 years (range 30–89). Males were 66% (P) vs. 83% (O) of participants (*p* = 0.005). The offer was rated overall as good to very good by both groups, i.e., 1.6 (P) vs. 1.6 (O; *p* = 0.7) on a scale from 1 to 6 with 1 being the highest rating. In line with the lower interaction in the digital format, the possibility to ask questions was rated worse with 1.5 (P) vs. 2.8 (O; *p* = 0.003). When asked about the desired future event format, two-thirds of the users of the digital patient forum were in favor of a hybrid event on-site and online.

**Conclusion:**

Patient events are suitable means of communication for the public and are rated well by visitors. In particular, interaction with experts is highly valued. Face-to-face formats are associated with a high logistical effort and high costs, and their reach is limited. In the future, hybrid formats could be a reasonable alternative, as they combine the advantages of online and face-to-face formats.

## Hintergrund

Moderne Angebote zur Patienteninformation und die medizinische Aufklärung der Bevölkerung haben eine wachsende Bedeutung [9]. Sie erfüllen Bedürfnisse der Informationsgesellschaft und bereiten die Basis für eine gelingende Partizipation der Betroffenen [2, 14, 22, 26, 33].

**Figure 1:**
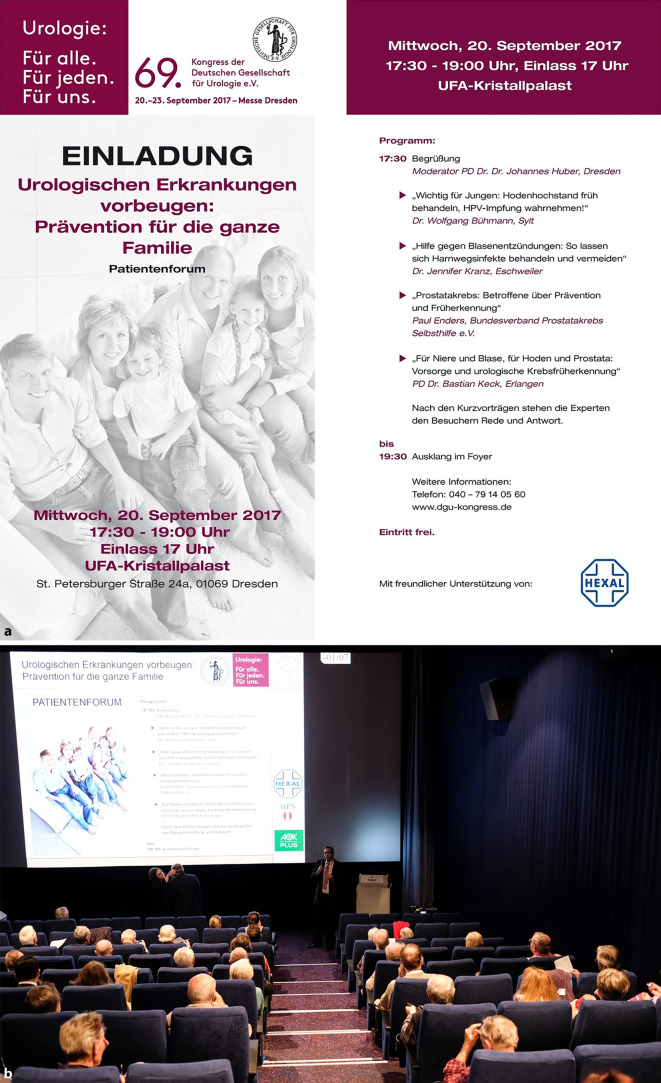

Durch das „Patientenforum“ setzt sich die Deutsche Gesellschaft für Urologie e. V. (DGU) auf ihren Jahreskongressen für die Patienteninformation und Öffentlichkeitsarbeit ein. In den letzten Jahren gestaltete sie unterschiedliche Formate – von laienverständlichen Frontalvorträgen in einem Kinosaal bis hin zu einer lockeren „Talkrunde mit Experten“ (Abb. 1). Diese Talkrunde wurde 2018 auch live online übertragen und die Veranstaltung 2019 für die anschließende Online-Nutzung aufgezeichnet (s. Tab. 3). Zuletzt erzwang die Coronapandemie eine vollständig digitale Umsetzung, sodass sich die Evaluationsergebnisse dieser vier Jahre hervorragend für eine vergleichende Bewertung eignen.

Ziel der Arbeit ist es, Präsenz- und Online-Veranstaltungen zu vergleichen und Schlussfolgerungen für die zukünftigen Inhalte und Formate zu ziehen. Welche Besucherkollektive können durch die Patientenforen angesprochen werden? Welche Themen sind für die Patient*innen interessant? Ergeben sich auch nach der Pandemiesituation Optimierungsmöglichkeiten für die Veranstaltungsorganisation? Dabei stellt sich die grundlegende Frage, ob aufwendige Patienteninformationsveranstaltungen ausschließlich in Präsenz weiterhin noch angemessen sind oder ob niederschwellige, flexible Online-Angebote die Zukunft darstellen.

## Material und Methoden

**Figure 2:**
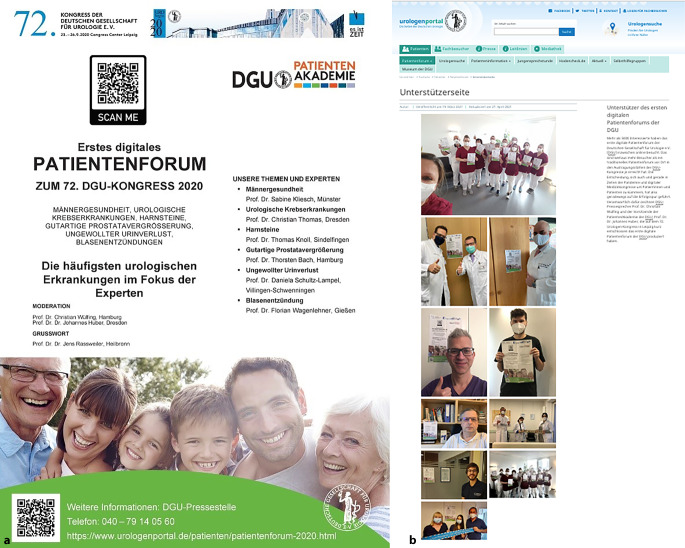

Für diesen Beitrag wurden die Präsenzpatientenforen des 69.–71. DGU-Jahreskongresses in Dresden und Hamburg (2017–2019) mit dem ersten digitalen Patientenforum 2020 verglichen (Abb. 1 und 2). Coronabedingt fand der 72. DGU-Kongress als CME-zertifiziertes „Best-of DGU 2020“ vom 24. bis 26. September 2020 in einem „Hybridformat“ statt (Abb. 2a). Zur Evaluation erhielten die Teilnehmenden einen zweiseitigen Fragebogen oder wurden zur Online-Evaluation via SurveyMonkey® (www.surveymonkey.com, SurveyMonkey Inc., San Mateo, CA, USA) aufgefordert. Da die gesamte Datenerhebung anonym erfolgte, war kein Ethikvotum erforderlich.

### DGU-Patientenforen

Die DGU organisiert anlässlich ihrer Jahreskongresse kostenlose Patientenforen zu populären urologischen Themen (Tab. 3). Hier können Expert*innen aus Kliniken und Praxen in verständlicher Sprache [33] über besondere oder häufige urologische Krankheitsbilder und Behandlungsmethoden berichten und Besucherfragen beantworten. Die Veranstaltungen wurden im Vorfeld in verschiedenen Publikationsformaten umfassend beworben: Beispielsweise erhielten 2018 etwa 120 urologische Facharztpraxen im 50-km-Radius um den Veranstaltungsort Dresden Einladungsmaterial für Patienten (Flyer und Poster Abb. 2). 2020 wurden deutschlandweit 5000 Einladungsposter als Einlage in der Fachzeitschrift *Urologie* verteilt.

### Fragebogen

Wir erstellten einen themenspezifischen Fragebogen, der aus Auswahlfragen und drei offenen Fragen bestand. Zu Beginn erhoben 11 Auswahlfragen die Aspekte Verständlichkeit, Themenauswahl, Informationsgehalt, Spaß, zeitlicher Rahmen, Veranstaltungsort, Organisation, Interaktion (Raum für eigene Fragen), Weiterempfehlung und Gesamtzufriedenheit auf einer Likert-Skala von 1 = „trifft vollständig zu“ bis 6 = „trifft gar nicht zu“. Anschließend wurde die Gesamtbewertung der Veranstaltung als „persönliche Schulnote“ erfragt (1 = sehr gut bis 6 = ungenügend). Außerdem wurden mit zwei Fragen Geschlecht und Alter der Teilnehmenden erhoben. In den drei Freitextfragen war Raum für Anregungen, Verbesserungsvorschläge und Themenwünsche für künftige Angebote (Tab. 4). Weiterhin erfassten wir, wie Teilnehmende von der Veranstaltung erfahren hatten, mit wem sie das Forum besuchten und ob sie sich in urologischer Behandlung befanden.

### Auswertung

Das Ende der Datenerhebung für die Online-Zugriffe war der 07. September 2021. Die statistische Auswertung erfolgte mit IBM SPSS Statistics 25 (IBM, Armonk, NY, USA) über χ^2^- sowie Mann-Whitney-U-Tests. Das Signifikanzniveau wurde auf α = 0,05 festgesetzt.

## Ergebnisse

Einen Überblick zu den Veranstaltungen und der Evaluation gibt Tab. 1. Insgesamt füllten *n* = 71 Besucher*innen der Präsenzpatientenforen der DGU-Jahreskongresse 2017–2019 die Fragebögen vollständig aus. Das Online-Patientenforum 2020 wurde von 18 Teilnehmenden bewertet. Ab dem Jahr 2018 wurde das Patientenforum zusätzlich als Videos aufgezeichnet und über das Urologenportal zugänglich gemacht. Nach Gründung der Urologischen Stiftung Gesundheit gGmbH (USG) sind die Patientenforen nun auf der Homepage der USG verfügbar (https://urologische-stiftung-gesundheit.de/patientenforen/). Für die Online-Inhalte zeigte sich eine zunehmende Resonanz: Die Anzahl der Videoaufrufe vervielfachte sich von 1207 (2018) über 2023 (2019) auf 7583 (2020).

**Table 1:** 

	2017	2018	2019	2020
Ort	Dresden	Dresden	Hamburg	Leipzig
Format	Kurzvorträge	Interaktiver Expertentalk (live im Internet)	Expertentalk und prominente Gäste	Erstes digitales DGU-Patientenforum
Titel	„Urologischen Erkrankungen vorbeugen: Prävention für die ganze Familie“	„Keine Scheu vorm Urologen“	„Prostatakrebs, Inkontinenz und HPV-Impfung“	„Die häufigsten urologischen Erkrankungen im Fokus der Experten“
Link zur Aufzeichnung	–	https://vimeo.com/292496405	https://player.vimeo.com/video/361995374	https://www.urologenportal.de/patienten/patientenforum-2020-1.html
Anzahl Seitenaufrufe Videos	–	1207	2023	7583
Medianes Alter	64	65	72	59
Fragebogenrücklauf	37	23	11	18
Weiblich	13 (38 %)	7 (30 %)	3 (27 %)	2 (11 %)
Männlich	24 (71 %)	16 (70 %)	8 (73 %)	13 (72 %)
Sponsoring	Firma Hexal AG	DGU	DGU	DGU

*HPV* humane Papillomviren

### Eigenschaften der Teilnehmenden

Die soziodemographischen Angaben pro Veranstaltung sind in Tab. 3 aufgeführt, der Vergleich zwischen den Formaten in den Tab. 1 und 2.

**Table 2:** 

Niere	Nierentumoren
	Nierenversagen
	Nephrolithiasis
Prostata	Behandlung des metastasierten Prostatakarzinoms
	Benigne Prostatahyperplasie
Komplementärmedizin	Alternativen zu schulmedizinischen Therapien (Naturheilkunde, Homöopathie)
	Stärkung des Immunsystems
	Selbstheilung, Gesundheitsfürsorge
Sonstiges	Erektile Dysfunktion
	Harninkontinenz
	Interstitielle Zystitis
	Kinderurologie
	Neue Diagnose- und Therapiemöglichkeiten
	Urologische Pflege für Angehörige

**Table 3:** 

	Präsenzveranstaltungen *n* = 71 (%)	Online-Veranstaltung *n* = 18 (%)	*p*-Wert
*Bestand im Vorfeld eine urologische Betreuung?*			0,5
… in urologischer Behandlung	31 (44)	7 (41)	
… in urologischer Vorsorge	16 (23)	2 (12)	
… bisher kein Kontakt	24 (34)	8 (47)	
*Wie wurde die Veranstaltung besucht?*			0,2
Besuch allein	44 (67)	15 (83)	
Besuch mit Partner:in	11 (17)	3 (17)	
Besuch mit Freunden	11 (17)	0 (0)	
*Wie haben Sie von der Veranstaltung erfahren?* *(Mehrfachantwort möglich; daher kein p‑Wert über alle Kategorien möglich)*			*n*/a
Internet	7 (9)	4 (25)	
Tageszeitung	18 (24)	0 (0)	
Einladungsflyer/Poster	23 (30)	2 (13)	
Persönliche Empfehlung	16 (21)	8 (50)	
Anderes	12 (16)	2 (13)	

Das mediane Alter der Teilnehmenden in den Präsenzveranstaltungen lag bei 66 (Range 30–89) vs. 59 Jahren in der Online-Gruppe (*p* = 0,7). Der Anteil männlicher Teilnehmer betrug in den Präsenzveranstaltungen 66 % vs. 87 % im digitalen Forumsformat (*p* = 0,005).

Die Tab. 1 zeigt die Ergebnisse für die folgenden drei Aspekte: Hinsichtlich der bereits vor dem Patientenforum bestehenden urologischen Versorgung zeigte sich kein signifikanter Unterschied (*p* = 0,5). Während die Präsenzveranstaltung von 67 % der Teilnehmenden allein besucht wurde, waren es im Rahmen der Online-Veranstaltung 83 %. Über alle Antwortkategorien war dieser Unterschied nicht signifikant (*p* = 0,2). Abschließend finden sich die Informationsmaßnahmen, mit deren Hilfe im Vorfeld über das Patientenforum informiert wurde. Während in der Gruppe der Teilnehmenden der Präsenzveranstaltung lediglich 21 % über persönliche Empfehlung auf die Veranstaltung aufmerksam wurden, waren es bei der Online-Veranstaltung mit 50 % die Hälfte (Mehrfachantwort, daher kein *p*-Wert über alle Kategorien möglich).

### Evaluation

Das Präsenzforum wurde insgesamt gut bewertet (1,6), wobei die Patientenforen 2018 (1,3) und 2017 (1,6) in Dresden die besten Bewertungen erhielten. Auch das digitale Forum wurde insgesamt gut bewertet (1,6). Tab. 2 zeigt die durchschnittlichen Bewertungen der Präsenzveranstaltungen gegenüber dem rein digital erfolgten Patientenforum. Bei nahezu allen erfragten Aspekten ergaben sich nahezu identische Bewertungen. Lediglich die Bewertung der Möglichkeit, Fragen zu stellen, weicht innerhalb der Befragten der Präsenzveranstaltungen und der Online-Veranstaltung deutlich voneinander ab (1,5 vs. 2,8; *p* = 0,003).

Positiv hervorgehoben wurden insbesondere die Allgemeinverständlichkeit der Talkrunde mit Expert*innen, die Themenauswahl sowie die gute Moderation. Kritisiert wurden die knapp bemessene Veranstaltungszeit und die geringe Besucherzahl. Als Themen für zukünftige Veranstaltungen wurden neben klassischen urologischen Themen auch die Berücksichtigung der Komplementärmedizin gewünscht. Außerdem wurden urologische Themenschwerpunkte speziell für Frauen und Kinder, Informationen zu neuen Therapien und Diagnosemöglichkeiten sowie urologische Pflege für Angehörige erbeten (Tab. 4).

**Table 4:** 

	Präsenzveranstaltungen (*n* = 71)	Online-Veranstaltung (*n* = 18)	*p*-Wert
Die Beiträge waren gut verständlich	Sehr gut (1,3)	Sehr gut (1,2)	0,08
Die Auswahl der Themen hat mir gefallen	Gut (1,6)	Gut (1,7)	1,0
Ich habe wichtige neue Informationen erhalten	Gut (2,0)	Gut (2,0)	0,9
Die Veranstaltung hat mir Spaß gemacht	Gut (1,9)	Gut (1,9)	0,4
Die Inhalte wurden verständlich erklärt	Sehr gut (1,5)	Sehr gut (1,2)	0,3
Der zeitliche Rahmen war angemessen	Gut (1,6)	Gut (1,8)	0,4
Der Veranstaltungsort/die Internetpräsenz hat mir gefallen	Gut (1,6)	Gut (1,7)	0,4
Die Organisation der Veranstaltung entsprach meinen Vorstellungen	Gut (1,7)	Gut (1,6)	1,0
Ich konnte meine Fragen stellen	Sehr gut (1,5)	Gut (2,8)	0,003
Ich würde die Veranstaltung weiterempfehlen	Sehr gut (1,4)	Sehr gut (1,5)	0,6
Ich bin mit der Veranstaltung insgesamt zufrieden	Gut (1,6)	Gut (1,6)	0,7

## Diskussion

Unser Vergleich der Evaluationsergebnisse der DGU-Patientenforen der Jahre 2017–2019 mit dem Online-Angebot des Pandemiejahres 2020 zeigt viele Parallelen hinsichtlich der Patientenwahrnehmung und -zufriedenheit, aber auch einige Unterschiede. Beide Veranstaltungsarten wurden überwiegend von Männern besucht, wobei der Anteil männlicher Teilnehmer im digitalen Format noch deutlich höher war. Auch hinsichtlich der Begleitung zeigten sich Unterschiede: Während die Präsenzforen sowohl allein als auch in Begleitung der Partner*in oder Freund*innen erfolgte, nahmen an der digitalen Veranstaltung mit 83 % ein höherer Anteil der Befragten allein teil. Dass sich über die Hälfte der Teilnehmenden zum Zeitpunkt der Befragung in urologischer Betreuung befand, zeigt ein starkes Interesse in dieser selbst betroffenen Bevölkerungsgruppe. Auf der anderen Seite erscheinen nicht betroffene Personen deutlich schwerer erreichbar zu sein. Die Patientenforen wurden insgesamt sehr gut bewertet, besonders gut gefiel den Patienten die Interaktion mit den Referenten bei den Präsenzformaten. Diese Möglichkeit zum direkten Austausch vermissten die Nutzer der Online-Veranstaltung. Hierdurch erklärt sich der signifikante Unterschied in der Bewertung der Möglichkeit, Fragen zu stellen (Note 1,5 vs. 2,8; *p* = 0,003). Auf die Gesamtzufriedenheit zeigte dies jedoch keinen messbaren Einfluss.

Die insgesamt sehr positive Bewertung des Forums auf den Jahreskongressen der DGU und im Online-Angebot belegt die Bedeutung von validen und verständlichen Informationsangeboten für Patienten. Während der Jahreskongress der DGU ein Fixpunkt im Kalender der deutschsprachigen urologischen Community ist, hat der Termin für Patient*innen und Laien keine Bedeutung. Gerade durch die wechselnden Veranstaltungsorte ist es extrem schwierig potenzielle Besucher zu erreichen und zum Besuch zu motivieren. In Bezug auf die Organisation von Patientenveranstaltungen ergeben sich daraus sehr große Herausforderungen, die gemessen an dem Gesamtaufwand die relativ niedrigen Besucherzahlen von etwa 20–70 Teilnehmenden an den Präsenzveranstaltungen erklären. Die geringe Besucherzahl bzw. Resonanz auf das Angebot wurde auch von den Teilnehmenden selbst kritisiert.

Der Kernpunkt zur Erhöhung der Bekanntheit und Reichweite muss folglich in patientenadäquater und zielgruppenrelevanter Werbung liegen. Hiermit könnten Patientenforen ein etablierter Bestandteil der Fachkongresse werden und im Sinne der Aufgaben einer Fachgesellschaft dem Informationsbedürfnis der Patienten besser gerecht werden. Diese Werbemaßnahmen machten auch in den evaluierten Jahren den weitaus größten Anteil der Kosten der Präsenzveranstaltung aus, wobei das Budget pro Präsenzveranstaltung etwa 4500 € betrug. Alle an der Organisation Beteiligten und alle Referent*innen engagierten sich dabei stets ehrenamtlich. Bei dem extrem schlechten „Wirkungsgrad“ von etwa 100 € Kosten pro Teilnehmenden ist die Aufzeichnung und Online-(Nach)nutzung der Veranstaltung nicht zuletzt auch ein Gebot der Wirtschaftlichkeit! Bezogen auf die während der Datenerhebungsphase erreichten Online-Aufrufe sinken die Kosten pro Teilnehmenden zumindest auf unter 1 €.

Die Evidenz zu Patientenveranstaltungen und speziell zum Vergleich von Online- und Präsenzformaten ist bislang unzureichend. Bekannt ist zumindest, dass das Interesse an Online-Informationen zu urologischen Entitäten zugenommen hat [19, 21]. Unsere Arbeitsgruppe hat vor einigen Jahren systematisch das Verhältnis von akademischem und öffentlichem Interesse an urologischen Themen analysiert und dabei die besondere Bedeutung andrologischer Themen sowie des Prostatakarzinoms herausarbeiten können [4]. Diese Schwerpunkte ließen sich auch bei der Evaluation des Urologenportals nachvollziehen [1]. Zum Vergleich von Online- und Präsenzformaten in der Prostatakrebsselbsthilfe konnten wir in einer großen Erhebung plausible Unterschiede identifizieren, die zumindest teilweise übertragbar erscheinen [16]: So dürften Online-Nutzer auch bei Informationsangeboten im Durchschnitt eher jünger und besser gebildet sein. In den Erziehungswissenschaften gibt es eine etwas belastbarere Evidenzlage zum Übergang vom Unterricht in Präsenzformaten zum teilweise oder vollständig online stattfindenden Unterricht [10, 20, 24]. Dabei gibt es vereinzelt Versuche, Erkenntnisse aus der pädagogischen Forschung auf die Informationsvermittlung und -schulung von Patienten zu übertragen [27, 30]. Die Auswirkungen der Coronapandemie auf den Online- und Präsenzunterricht wurden dabei vielfach untersucht, jedoch erscheint die Qualität von einigen sehr rasch initiierten Studien zumindest fraglich [11].

Die Grundmotivation für ein intensiviertes Informationsangebot der DGU speist sich aus der gestiegenen Relevanz von leitliniengerechten Informationen für Patient*Innen, mit dem Ziel an partizipativer Entscheidungsfindung teilhaben zu können [5, 6, 13, 25]. Aufgrund veränderter Informationsgewohnheiten nutzen Betroffene immer stärker Angebote aus dem Internet [1, 4, 13, 16, 17, 28, 29, 35], da sie einen sehr niederschwelligen Zugang ermöglichen. Aufgrund dieses immer heterogeneren Informationsspektrums haben valide und evidenzbasierte Angebote, wie etwa das Patientenforum der DGU, direkte und positive Effekte auf die Patienteninformation [8, 18]. So können sie den Patienten helfen, komplexe medizinischen Themen besser zu verstehen [3, 7] und partizipatorische Ansätze bei der Therapieentscheidung unterstützen [23, 31, 32, 34]. Zur Verbesserung der Regelversorgung und Stärkung der Patientenrolle eignen sich insbesondere neue digitale Gesundheitsangebote, deren Potenzial in der deutschen Urologie immer noch zu wenig genutzt wird [12, 15].

Bei der Überarbeitung und Optimierung des Formats des Patientenforums sollte folglich entsprechend unserer Ergebnisse geprüft werden, ob Hybridformate eine sinnvolle Alternative darstellen können. Präsenzveranstaltungen bieten unumstritten Vorteile in der direkten Kommunikation, sind allerdings mit hohen Kosten und einem nicht großen logistischen Aufwand für das Publikum und die Veranstalter verbunden. Um den Rezipientenkreis der Patientenforen zukünftig zu erhöhen, kann eine stärkere Nutzung von Angeboten moderner, onlinebasierter Kommunikationsmittel (u. a. Social Media) hilfreich sein.

Zudem lässt sich so auch eine jüngere, interessierte Zielgruppe ansprechen. Ein Beispiel hierfür sind die Angebote im Rahmen der DGU-Themenwochen. Online-Angebote stellen eine sinnvolle und nützliche Ergänzung etablierter Formate dar [16], da sie es erlauben, die Veranstaltung auf bekannten Streaming-Plattformen oder im Nachhinein als Video zu verfolgen. Zudem ist die Teilnahme für interessierte Patienten niederschwelliger möglich als bei Präsenzveranstaltungen.

**Figure 3:**
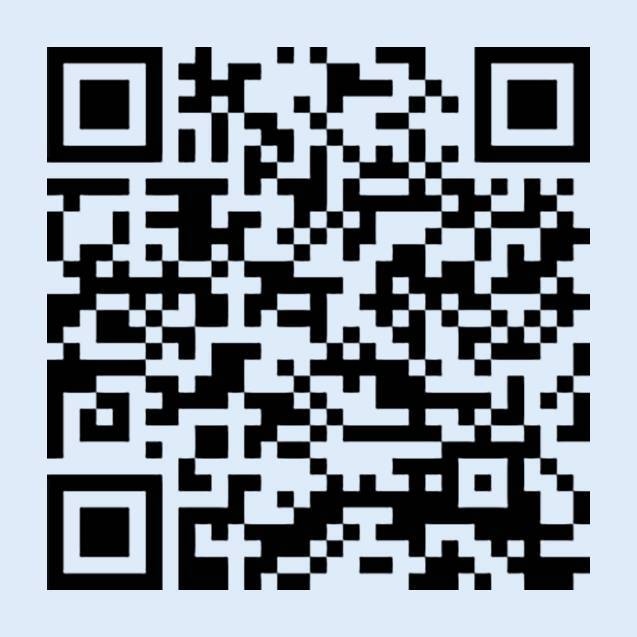

Zur Intensivierung der Kommunikation urologischer Themen in die Öffentlichkeit gründete die DGU im Jahr 2021 die Urologische Stiftung Gesundheit gGmbH. Die Stiftung baut schrittweise ein Informations- und Serviceportal für Betroffene und interessierte Laien auf und hält hochwertige Unterstützungsangebote vor, wie die Entscheidungshilfe Prostatakrebs (www.entscheidungshilfe-prostatakrebs.info). Wichtige Schwerpunkte der Stiftung sind außerdem die Patienteninformation und die Vermittlung von Wissen zur urologischen Gesundheitsfürsorge und -förderung. Auch die bisherigen Patientenforen sind auf der neuen Online-Präsenz zu finden (Abb. 3). Dadurch übernimmt die Stiftung die bisherige Informationsfunktion des Urologenportals für interessierte Laien (www.urologenportal.de; [2]), sodass sich das Urologenportal künftig auf das Fachpublikum fokussieren kann.

### Stärken und Einschränkungen

Diese Evaluation bildet eine gute Grundlage für die Weiterentwicklung des Patientenforums. Ihre Ergebnisse erlauben es, auf die spezifischen Bedürfnisse der Teilnehmer besser einzugehen und gewünschte Inhalte ausführlicher zu berücksichtigen. Die Ergebnisse geben einen guten Eindruck von den Interessen und Bedürfnissen (potenzieller) urologischer Patienten, ihrer Angehörigen, Bekannten/Freunden und anderer Personen beispielsweise aus dem Bereich der Selbsthilfe.

Die vorliegenden Ergebnisse unterliegen einer Reihe von Limitationen: Der genutzte Fragebogen wurde keiner formalen Validierung unterzogen. Durch die Nutzung einer gut etablierten Likert-Skala und den Bezug auf Schulnoten war jedoch eine hohe Augenscheinvalidität („face validity“) gegeben. Aufgrund des zahlmäßig geringen Besucherkollektivs fällt die Stichprobe nur klein aus. Zudem beteiligten sich nicht alle Besucher an der Evaluation („selection bias“). Bei der Evaluation kann auch eine Stichprobenverzerrung bei den Antworten durch die soziale Erwünschtheit eine Rolle spielen, wogegen allerdings die Tatsache spricht, dass die Teilnahme anonym erfolgte. In jedem Fall erlauben online durchgeführte Patientenforen prinzipiell eine einfachere Evaluation, da sie unmittelbar digital erfolgen kann. Um so bedauerlicher ist es, dass der Rücklauf in der Online-Gruppe mit 18 Evaluationen bei 7583 Seitenaufrufen sehr gering ausfiel. Dieser Rücklauf von formal 0,2 % stellt sicher die stärkste methodische Limitation dar. Diese starke Selektion erklärt auch die hohe Relevanz der persönlichen Weiterempfehlung in diesem Kollektiv. Nach persönlicher Empfehlung aufmerksam gewordene Teilnehmende waren sicher stärker für einen Abschluss der Evaluation motiviert.

## Fazit für die Praxis

Patientenveranstaltungen werden sehr geschätzt und die Interaktion mit Expert:innen hat einen hohen Stellenwert. Präsenzformate sind mit einem hohen logistischen Aufwand verbunden und von limitierter Reichweite; sie erfreuen sich aber als etabliertes Format hoher Beliebtheit bei einem älteren Patientenklientel. Online-Formate dagegen zeigen ein hohes Entwicklungspotential. Sie erfordern jedoch technischen Support und Know-how, v. a. beim Einbezug der virtuellen Besucher*innen. Hier wird der Anteil an Social-Media-Angebote und der Anteil an Interaktion mit den Usern/Besuchern noch steigen. Zukünftig können Hybridformate eine sinnvolle Alternative sein, da sie die Vorteile von Online- und Präsenzformaten kombinieren. In jedem Fall sollten die Patientenforen weiterhin live aufgezeichnet werden und als kostenlose Videos zur Verfügung gestellt werden, da die an Seitenaufrufen gemessene Resonanz hoch ist und sich erhebliche Gestaltungspotentiale für die Zukunft andeuten.

## Abkürzungen

DGU: Deutsche Gesellschaft für Urologie e. V.
HPV: Humane Papillomviren
O: Onlinegruppe/Besucher*innen des Online-Patientenforums
P: Präsenzgruppe/Besucher*innen der Präsenzpatientenforen
USG: Urologische Stiftung Gesundheit gGmbH
